# 

**Published:** 1898-06

**Authors:** 


					﻿VOL. XVIII.
JUNE, 1898.
NO. 6?
' ’sirRorl
THE
JIOMCOPATHIC pHY^lClA^,
A Monthly Journal of Homoeopathic Materia Medica
and Clinical Medicine.
“ He i» a freeman whom truth makes free, and all are slaves beside."
"Seek the Truth: come whence it may, cost what it will."
EDITED AND PUBLISHED BY
WALTER M. TAMES. ML D.
PHILADELPHIA:
No. 1231 LOCUST STREET.
T~ Yearly Subscription, $2.50, invariably in advance. Single numbers, 25 els.
Entered at the Philadelphia Poit-OtBoe as Seeond-Class Mail Matter.
				

